# Healthcare professionals’ perceptions on the emotional impact of having an inadequate response to antidepressant medications: survey and prospective patient audit

**DOI:** 10.1186/s12991-018-0189-z

**Published:** 2018-05-11

**Authors:** Rajnish Mago, Andrea Fagiolini, Emmanuelle Weiller, Catherine Weiss

**Affiliations:** 1 Simple and Practical Mental Health, Philadelphia, PA USA; 2 University of Siena Medical Center, Siena, Italy; 3 H. Lundbeck A/S, Valby, Denmark; 4 Otsuka Pharmaceutical Development & Commercialization, Inc, Princeton, NJ USA; 5 210 W Rittenhouse Square Suite 404, Philadelphia, PA 19103 USA

**Keywords:** Depression, Antidepressant, Frustration, Audit

## Abstract

**Background:**

Despite the availability of effective antidepressants, about half of patients with major depressive disorder (MDD) display an inadequate response to their initial treatment. A large patient survey recently reported that 29.8% of MDD patients experiencing an inadequate treatment response felt frustrated about their medication and 19.2% were frustrated with their healthcare provider. This survey and chart audit evaluated healthcare professionals’ (HCP) views on the emotional impact of having an inadequate response to antidepressant medication.

**Methods:**

HCPs who frequently treat patients with MDD completed a survey and chart audit of their MDD patients currently experiencing an inadequate response to antidepressant treatment.

**Results:**

287 HCPs completed 1336 chart audits. HCPs reported that 38% of their patients were trusting/accepting of their MDD medications and 41% of their patients trusted/felt confident with their healthcare provision. Conversely, HCPs reported that 11% of their patients were frustrated with their medication and 5% with their healthcare benefits. HCPs cited impact on daily life (53%) and treatment issues (lack of efficacy and side effects; 50%) as the main drivers for their patients’ feelings of frustration. When HCPs recognized patients’ feelings of frustration, the top concerns of the HCPs were worsening of symptoms (43%) and non-compliance (41%).

**Conclusions:**

This survey and chart audit highlights the emotional burden associated with inadequate responses to MDD treatment in addition to persistent symptoms. Differences between the views of the HCPs and patients are highlighted and suggest that HCPs may underestimate the full impact that having to try numerous medications has on their patients.

## Background

Despite the availability of many effective antidepressants, in about half of patients, major depressive disorder (MDD) responds inadequately to the initial treatment, leaving patients to cope with persistent symptoms while their medication plan is optimized [1–3]. Persistence of depressive symptoms is known to be associated with various adverse outcomes, including a greater risk of relapse and recurrence [4, 5], a shorter duration between episodes [4], continued impairment in work and relationships [6] and increased overall mortality from comorbid medical disorders [7–10].

We have recently reported the results of a large, international survey of 2096 patients with MDD which was designed to better understand the emotional impact of having an inadequate response to antidepressant medication [11]. The patient survey found that the most frequently reported emotion associated with an inadequate treatment response was ‘frustration’ (29.8% of respondents). This frustration was directed towards their medication and/or their HCP, and was cited as a cause of patients wanting to stop their medication. To build an effective therapeutic alliance and help patients better engage with their treatment plan, it is essential that HCPs understand the patient’s perspective. However, there is often a disconnect between the patient and HCP perceptions of depression management [12]. The aim of this HCP survey and chart audit was to evaluate HCP’s views on the emotional impact on the patient of having an inadequate response to antidepressant medication, and to compare these findings with the patient survey.

## Methods

This HCP survey and chart audit was conducted in the United States (US), Canada, United Kingdom (UK), Germany, France, and Spain between 14 March and 15 June 2016. No personal identifying information about any patient was requested and the audit was compliant with the European Pharmaceutical Market Research Association (EphMRA) and Association of the British Pharmaceutical Industry (ABPI) Codes of Conduct and all guidelines set forth by the Health Insurance Portability and Accountability Act (HIPAA). In line with the Data Protection Directive 95/46/EC, access to the online audit was secure and all relevant data was kept strictly confidential and anonymous. The authors designed the study and the survey and analyses were conducted by an independent market research agency (Market Strategies International, Livonia, MI, USA funded by Otsuka Pharmaceutical Development & Commercialization, Inc. and H. Lundbeck A/S).

### Participants

Respondents were recruited from a database of HCPs who have previously agreed to participate in research. HCP respondents had to be either a board certified/eligible psychiatrist or a primary care physician (PCP; US only) with a 3–35-year history of practicing adult psychiatry in the outpatient setting and currently spending at least 70% of time in direct patient care. All respondents were required to be seeing at least 20 patients with MDD per month. HCPs working in mental health had to be currently initiating prescriptions for MDD (any treatment) including atypical antipsychotics. Primary care physicians were required to be either initiating or refilling prescriptions (any medication) for the treatment of MDD.

### Study design

Respondents were blinded to the key study objectives, but were aware that the survey was designed to collect information on current MDD management with the aim of improving patient care. The study was carried out in two distinct parts. The first part was a survey about the HCP’s clinical practice, and for the second part, eligible respondents were asked to complete the chart audit for 5 outpatients with MDD who were still experiencing clinically significant depressive symptoms after at least 6 weeks of antidepressant treatment at the recommended dose. It was estimated that it would take 45 min to complete the audits over the course of a week (5–7 min per patient chart, preferably no more than one a day).

### Chart audit

To ensure adequate recall, HCP respondents were instructed to complete each patient audit within 8 h of seeing the patient and were encouraged to refer to the patient medical record for accuracy. Full inclusion criteria for suitable patients to be included in the audit are shown in Table 1.

**Table 1:** Patient criteria for inclusion in audit

Age 18–65 years old
Diagnosed with major depressive disorder (MDD) Has never had dysthymia No other comorbid psychiatric conditions (e.g., schizoaffective disorder, etc.)
Being treated in an outpatient setting
Experiencing an MDD episode that required prescription treatment
Treated with an antidepressant at the recommended dose for at least 6 weeks who still experiences clinically significant depressive symptoms
Has been taking prescription medication under your care for at least 3 months for their current episode of MDD and whom you are seeing/treating in a follow-up visit

The chart audit included up to 31 items (dependent on responses) and was structured to collect information on: patient characteristics, treatment history, clinical evaluation, HCP perceptions of patient’s emotions associated with an inadequate response to treatment and the HCP respondent’s perception of whether their patients experience ‘frustration’ with aspects of their healthcare. As part of the patient characteristics section, HCP respondents were asked to rate their patient’s functional ability using the Sheehan Disability Scale (SDS) [13] where a score of 0 represents no impact of symptoms on patient function and 10 represents ‘extreme’ disruption.

To assess how HCPs recognize patient feelings of frustration and dissatisfaction, respondents were first prompted to indicate how they believed their patients felt about their healthcare, choosing from a list of 14 multi-choice answers. Feelings of frustration and dissatisfaction were included as two of the 14 items. The 14 items were: understood, anonymous, frustrated, dissatisfied, neglected, confused, impatient/irritated, apprehensive, hopeless/doubtful, unimportant, ignored, trapped/helpless, none of the above and I am not able to answer this question. If the HCP respondent identified feelings of either frustration or dissatisfaction in their patients, the next set of questions explored which aspects of healthcare they believed the patients were frustrated/dissatisfied with. Further questions included potential sources for frustration/dissatisfaction, and the impact of these feelings.

### Data analysis

All HCP responses were coded and analyzed using descriptive statistics (means and frequency of responses).

## Results

### Sample

A total of 1300 HCPs were screened for inclusion in this survey. Of these, 287 met MDD practice criteria and agreed to the study requirements. Overall, 287 HCPs completed a total of 1336 patient chart audits, and of these 256 HCPs completed all 5 charts. Tables 2 and 3 describe the HCP and patient characteristics, respectively. Overall, 38% of patient charts were from the US, and the rest from the UK, France, Germany, Spain and Canada. Of the 513 US patient charts, half (*n* = 254 or 19% of all charts) were completed by PCPs; all other patient charts were completed by psychiatrists.

**Table 2:** Characteristics of the HCP respondents

Variable	*N* = 287
HCP specialty; *n* (%)	
Psychiatrist	234 (82%)
US PCP	44 (15%)
Internist/internal medicine	7 (2%)
Nurse practitioner	2 (1%)
Mean years in practice [range]	17.0 years [3–35 years]
Mean percent of time spent in direct patient care	92%
Median estimated numbers of patients seen per month	
MDD	70
Bipolar disorders	30
Schizophrenia	30
Schizoaffective disorder	15
HCP location; *n* (%)	
US	108 (38%)
Canada	36 (13%)
UK	40 (14%)
Germany	35 (12%)
France	37 (13%)
Spain	31 (11%)
Setting	
Outpatient	251 (88%)
Inpatient	36 (13%)

**Table 3:** Patient characteristics per chart audit

Variable	Statistic *N* = 1336
Setting	
Office	802 (60%)
(US only) Outpatient Community Health Clinic	75 (6%)
(France only) Centre Medical Psychologique	53 (4%)
Hospital Outpatient Clinic	351 (26%)
Telemedicine	12 (1%)
Patient’s Home	23 (2%)
Day Clinic	19 (1%)
Other	1 (< 1%)
Length of MDD diagnosis	
< 1 month	88 (7%)
1–3 months	174 (13%)
4–6 months	180 (14%)
7–9 months	65 (5%)
10–12 months	211 (16%)
2–5 years	370 (28%)
6 + years	248 (19%)
Current treatment	
SSRI	800 (60%)
SNRI	320 (24%)
MAOI	7 (< 1%)
TCA	49 (4%)
Other antidepressant	295 (22%)
Anxiolytic	239 (18%)
Antipsychotic	243 (18%)
Hypnotic	76 (6%)
Other treatment for depression	69 (5%)
Mean current ADT duration by treatment class (weeks)	
SSRI	45.4
SNRI	48.7
MAOI	22.0
TCA	106.8
Other antidepressant	38.9
Number of current classes of prescription treatments for depression (this episode); n (%)	
0	6 (< 1%)
1	755 (57%)
2	397 (30%)
3	145 (11%)
4	28 (2%)
5 +	5 (< 1%)
Mean number	1.6
Level of functioning (mean SDS scores)	
SDS Mean score	5.1
Work domain	6.0
Social domain	4.7
Home domain	4.5
PHQ-9 score (mean)	6.2
Clinical global impression of change in depression since onset of episode to current visit	
Very much worse	5 (< 1%)
Much worse	63 (5%)
Minimally worse	113 (9%)
No change	248 (19%)
Minimally improved	413 (31%)
Much improved	400 (30%)
Very much improved	94 (7%)

### Goal setting

Overall, HCPs reported discussing treatment goals with 1189 (89%) of their patients. Of these, most (*n* = 1089; 92%) said their patients agreed with the set goals and only 100 (8%) patients disagreed with the goals. From the HCP perspective, the main goals of treatment were ‘improve symptoms of depression’ (54.8%), ‘improve social functioning’ (44%), ‘improve symptoms of anxiety’ (41%), ‘engage in social activities’ (39%), ‘reach remission and eventually be treatment free’ (30%), ‘improve sleep problems’ (30%), ‘improve cognitive symptoms’ (28%), ‘be able to be productive at work’ (27%) and ‘be able to better manage home duties’ (23%).

### Emotions associated with an inadequate response to treatment

**Fig. 1:**
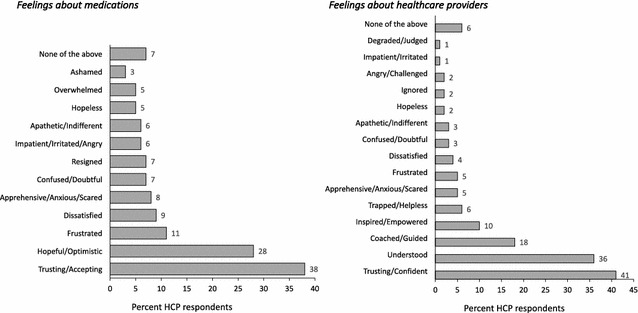
HCP perceptions of how patients with MDD and experiencing treatment failures feel about **a** medications, **b** their healthcare provider

Per HCP report, a majority of patients were trusting/accepting (38%) of their medications for MDD and a similar proportion trusted/felt confident (41%) about their healthcare provider. Conversely, HCPs reported that about one in ten of their patients were frustrated (11%) and/or dissatisfied (9%) with their medication and one in twenty patients were frustrated (5%) and/or dissatisfied with the healthcare provider (Fig. 1).

Further analysis revealed that feelings of frustration and dissatisfaction with medication were identified significantly more frequently by HCPs in patients with a longer history of MDD or who had more antidepressant failures. For example, HCPs identified frustration in 10.7% of patients with a 10-year history of depression (*n* = 22 of 205 patients) compared to 4.5% in patients with a 2-year history (*n* = 6 of 134 patients). Likewise, HCPs identified feelings of frustration with medication in 7.3% of patients who had experienced 3 or more antidepressant treatment failures in the current episode (*n* = 48 of 660 patients) versus 1.8% in patients who had experienced two treatment failures (*n* = 7 of 385 patients) and 2.7% in patients with one treatment failure (*n* = 8 of 291 patients). HCPs were also more likely to identify that their patients were frustrated with healthcare in patients who they had classed (using the SDS scale) as having severe disruption to their daily life (frustration with healthcare was identified in 3.8% of severely affected patients vs. 1.5% of mildly affected patients). Of note, HCPs practicing in Spain and France reported that fewer of their patients were frustrated with medication and/or healthcare (6 and 8%, respectively) compared with the USA (15%), Germany (12%), UK (17%) and Canada (17%).

### Drivers and consequences of frustration in MDD

**Fig. 2:**
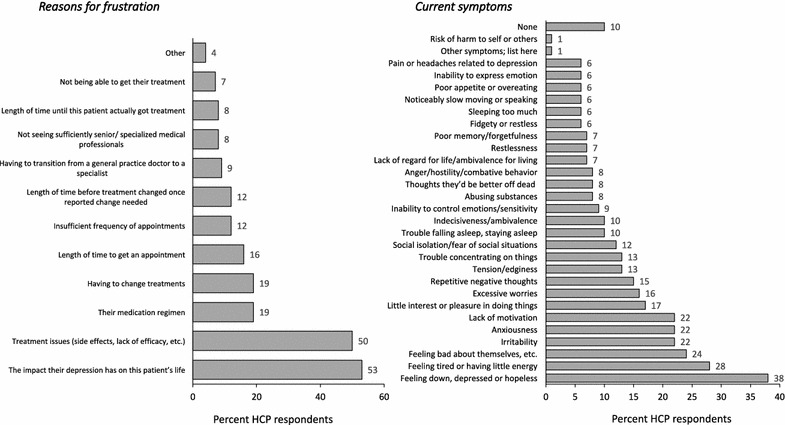
HCP perceived reasons for patient frustration with overall healthcare **a** overall, **b** current symptoms

When the HCP identified that their patients had feelings of frustration or dissatisfaction with healthcare (*n* = 156 patients), the main drivers of frustration were thought to be: impact on daily life (53%) and treatment issues (i.e., lack of efficacy and side effects; 50%). HCPs considered the medication regimen and having to change treatments as less important (both were considered a driver in 19% of patients) (Fig. 2a). HCPs identified that a wide range of symptoms may be related to frustration with healthcare, the most common being ‘feeling down, depressed or hopeless’ (38%) and feeling tired or having little energy (28%) (Fig. 2b).

**Fig. 3:**
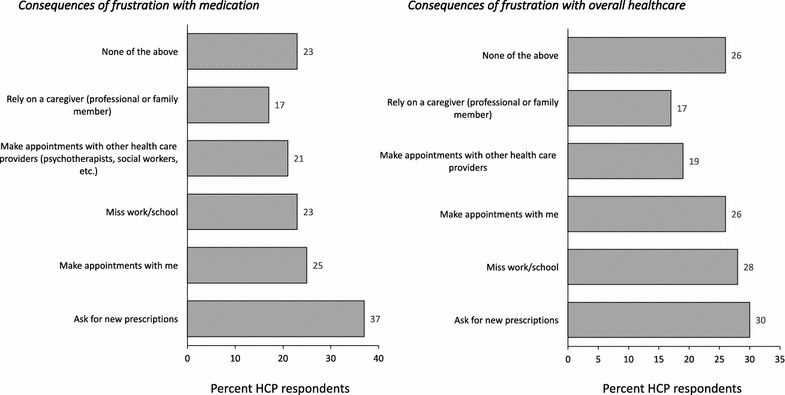
HCP perceived consequences of patient frustration with **a** medication, **b** overall healthcare

In those patients recognized to be frustrated with their medication (*n* = 149), HCPs indicated that they are most likely to react by asking for a new prescription (37%), to make a new appointment (25%) and/or missing school (23%) (Fig. 3a). Likewise, in those patients believed to be frustrated with overall healthcare (n = 156), HCPs indicated that they were more likely to ask for a new prescription, but also to miss school or work and ask to see the doctor more frequently (Fig. 3b).

**Fig. 4:**
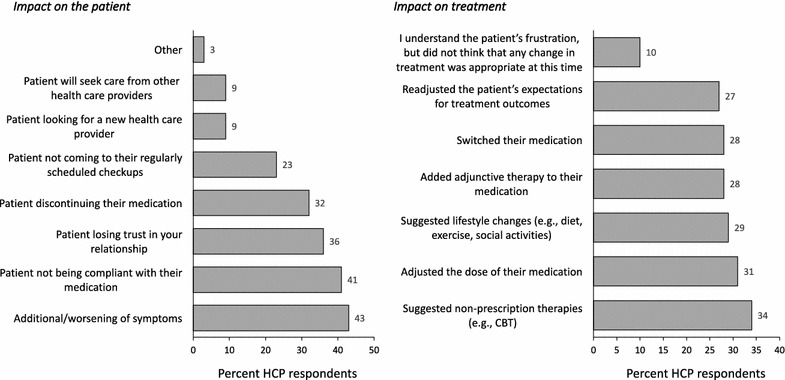
Impact of frustration **a** concerns about impact on the patient, **b** impact on treatment decisions

When HCPs recognized feelings of frustration in their patient, the top concerns were: worsening of symptoms (43%) and non-compliance (41%) (Fig. 4a). HCPs most commonly suggested non-pharmacological therapies (e.g., cognitive behavioral therapy), adjustment of medication doses, and/or lifestyle changes (Fig. 4b).

## Discussion

To the best of our knowledge, this survey is the first to evaluate how HCPs perceive the emotional impact of inadequate response to antidepressant treatment on their patients. Although HCPs reported that a large percentage of their patients experiencing inadequate response to antidepressant treatment had positive feelings about their medication and healthcare, they also identified a range of negative emotions including frustration and dissatisfaction, both with medications and overall healthcare. Such emotions are important to address, as they can directly impact medication adherence. Indeed, non-compliance was one of the top concerns HCPs associated with patient frustration.

The results of this survey indicate that HCPs are aware of the wide range of negative emotions that can potentially be associated with treatment failure and persistent depressive symptoms. No HCP reported that all of their patients included in the audit had only positive feelings towards their medication and/or healthcare. However, comparing the results of this survey to a recent patient survey, it appears that HCPs may significantly underestimate how many of their own patients have negative emotions towards their medication or healthcare, and may not be identifying the full impact of treatment failures on the patient experience. Whereas HCPs identified feelings of frustration with medication in 11% of their patients, the self-reported level of frustration in the patient survey was nearly three times higher (30%). Only half of the HCP respondents recognized frustration in any of their patients. Likewise, whereas HCPs recognized that 12% of their patients are frustrated with their healthcare, the patient survey indicated that a much larger percentage (27%) are frustrated with their overall healthcare, including access to services and medications and experiences with their doctors, nurses and therapists.

Of note, HCPs only considered that 5% their patients are frustrated with their therapeutic relationship compared to 19% of patients in the patient survey. When HCPs did recognize feelings of frustration in their patients, they appeared to be aware of the potential impact of frustration on medication adherence and cited worsening of symptoms and non-adherence as their top concerns associated with frustration. However, it is notable that only 24% of HCPs identified patient frustration in the audit, and these HCPs usually identified it in 2 or 3 of their 5 patient charts. The lack of recognition from the 76% of HCPs likely accounts for the discrepancy between the HCP and patient surveys in the reported prevalence of frustration in this patient population. Recognizing frustration with the therapeutic alliance is vital to address, because 29% of patients in the patient survey reported that they share less information when they are frustrated with their HCP and 27% reported wanting to quit their medication altogether. This insight is supported by the findings of another study based on in-depth interviews which found that MDD patients who had stopped taking their antidepressants had often experienced unsatisfactory interactions with HCPs [14]. Other qualitative studies have identified patient ambiguity and frustration with their medication (including time-frame of treatment, efficacy and tolerability) as key reasons for medication non-adherence [15].

One practical way to improve the therapeutic alliance is to engage the patient in goal setting, so that they have reasonable expectations of their treatment. Moreover, it has been suggested that when patients receive treatment that they perceive as relevant to their individual needs, they are likely to exhibit greater commitment to their treatment regimen [16–18]. This, in turn, may help to significantly decrease discontinuation of treatment, increase satisfaction, and ultimately improve outcomes. In this survey, HCPs reported that they had discussed treatment goals with 90% of their patients. But in the patient survey, the proportion of patients who said treatment goals had been discussed with their HCPs was smaller (72%). Effective goal setting requires effective communication between the HCP and patient and it is a therapeutic skill that needs to be learned and practiced [19]. It may also be that some patients in the patient survey did not explicitly realize or remember that their HCP had discussed the goals of treatment. Interestingly, while patients and HCPs both agreed that their top goal for MDD treatment was to address depressive symptoms (55% in the HCP survey and 78% in the patient survey), patients are more inclined to expect improvement in anxiety (61% in patient survey vs. 41% in HCP survey) and sleep issues (51% in patient survey vs. 30% in HCP survey) than HCPs.

Strengths of the study include its international design and the timing of the patient audit where respondents were asked to record the data soon after they had seen the patient (limiting recall bias). Limitations of the survey include all those inherent to survey methodology including the process of recruitment which was limited to a commercial database of HCPs. Although there was consistency of most results across countries, this survey indicated that there may be some national differences in the perception of frustration, with lower levels being reported in Germany and France. This may be because of cultural differences in the patient population and/or in the healthcare system organization for MDD; and indeed, the patient survey found lower levels of patients expressing frustration in France [11]. Studies at the national level may be better able to tease out what aspects of care lead to frustration in each population. In addition, since the patient and HCP surveys were conducted separately, and patients were not matched to the HCP, a limitation of the various comparisons discussed above is that we cannot rule out the possibility that there were inherent differences in the patient populations surveyed. Although both surveys were conducted in the same countries, the sample size of the HCP survey was smaller than the patient survey (*n* = 287 HCP/1336 patient charts vs. *n* = 2096 patients, respectively), and the relative representation of respondents from each country differed slightly (e.g., 38% of HCP respondents vs. 28.5% patient respondents were from the US). Moreover, it may be that patients experiencing frustration with their healthcare or medication are more likely to participate in a survey as a means to voice their dissatisfaction.

## Conclusions

This survey highlights the high prevalence of wide-ranging emotional burden associated with treatment failures in MDD. Although HCPs appear to be aware of some of the problems, the discrepancies between the results of this HCP survey and the patient survey [11] suggest that HCPs may often underestimate the full impact of having to try numerous medications has on their patients. The results can be considered a ‘call to action’ for clinicians to consider their management approach for patients who show an inadequate response to antidepressant treatment. In particular, HCP awareness of how patients experience ‘frustration’ appears to be low compared to the self-reported prevalence in patients with MDD. This is important to consider because patients report that feelings of frustration may lead to poor adherence to medication, which will then continue to contribute to poor outcomes in patients.

## Abbreviations

ABPI: Association of the British Pharmaceutical Industry
EphMRA: European Pharmaceutical Market Research Association
HCP: healthcare professional
HIPAA: Health Insurance Portability and Accountability Act
MDD: major depressive disorder
PCP: primary care physician
